# Perspectives on Post-COVID-19 Pulmonary Fibrosis Treatment

**DOI:** 10.3390/jpm14010051

**Published:** 2023-12-29

**Authors:** Elena Cojocaru, Tudor Cojocaru, Giulia Mihaela Pînzariu, Ioana Vasiliu, Ioana Armașu, Cristian Cojocaru

**Affiliations:** 1 Morpho-Functional Sciences II Department, “Grigore T. Popa” University of Medicine and Pharmacy, 16 University Street, 700115 Iasi, Romania; elena.cojocaruu@umfiasi.ro (E.C.); ioana.vasiliu@umfiasi.ro (I.V.); 2 Faculty of Medicine, University of Medicine and Pharmacy, 16 University Street, 700115 Iasi, Romania; mg-rom-30246@students.umfiasi.ro (G.M.P.); ioana.armasu@umfiasi.ro (I.A.); 3 Medical III Department, “Grigore T. Popa” University of Medicine and Pharmacy, 700115 Iasi, Romania; cristian.cojocaru@umfiasi.ro

**Keywords:** pulmonary, fibrosis, post-COVID-19, treatment, antifibrotic, nintedanib, pirfenidone

## Abstract

Pulmonary fibrosis, a critical outcome of chronic inflammatory diseases, has gained prominence in the context of post-coronavirus (post-COVID-19) complications. This review delves into the multifaceted landscape of post-COVID-19 pulmonary fibrosis, elucidating the intricate molecular mechanisms underlying its pathogenesis and highlighting promising therapeutic avenues. Examining the aftermath of severe acute respiratory syndrome-2 (SARS-CoV-2) infection, the review reveals key signaling pathways implicated in the fibrotic cascade. Drawing parallels with previous coronavirus outbreaks enhances our understanding of the distinctive features of post-COVID-19 fibrosis. Antifibrotic drugs, like pirfenidone and nintedanib, take center stage; their mechanisms of action and potential applications in post-COVID-19 cases are thoroughly explored. Beyond the established treatments, this review investigates emerging therapeutic modalities, including anti-interleukin agents, immunosuppressants, and experimental compounds, like buloxybutide, saracatinib, sirolimus, and resveratrol. Emphasizing the critical importance of early intervention, this review highlights the dynamic nature of post-COVID-19 pulmonary fibrosis research. In conclusion, the synthesis of current knowledge offers a foundation for advancing our approaches to the prevention and treatment of these consequential sequelae of COVID-19.

## 1. Introduction

Since December 2019, humanity has entered a new era of respiratory infectious diseases. Before the current coronavirus (COVID-19) pandemic, two other major outbreaks occurred after 2000: severe acute respiratory syndrome related coronavirus (SARS-CoV) in 2002 and Middle East respiratory syndrome (MERS) in 2012 [1]. These epidemics, like the current pandemic, were caused by coronaviruses. However, none of these global public health challenges has prompted an adequate level of preparedness in healthcare systems, even in developed countries.

The World Health Organization proclaimed a Public Health Emergency of International Concern on 30 January 2020, and a pandemic on 11 March 2020 [2]. As of 13 December 2023, over 772 million confirmed cases and almost 7 million deaths were reported globally [3].

The COVID-19 pandemic, a global health crisis of unprecedented proportions, has left a lasting impact on public health and medicine. While considerable attention has rightly been devoted to understanding and combating the acute effects of the SARS-CoV-2 virus, there is a growing concern about the long-term health consequences experienced by those who survived the infection. Among these post-acute sequelae of SARS-CoV-2 infection, post-COVID-19 pulmonary fibrosis stands out as a significant and challenging condition [4].

According to the World Health Organization, most patients with COVID-19 completely recover, but the recent data indicate that 10–15% of patients experience a range of medium- and long-term side effects after the initial disease [5]. It is currently unclear who is more likely to develop post-COVID-19 fibrosis, although breathlessness appears to be more common in women and among those who initially had more severe COVID-19. A study found that 44.9% of COVID-19 survivors developed pulmonary fibrosis, significantly correlated with the development of post-COVID-19 pulmonary fibrosis [6].

The implementation of drastic measures and the initial lack of protective equipment reveal that humanity is not prepared for a disease with a global impact. Major concerns, including the high rate of intensive care unit admissions [7], the high mortality rate, and the unavailability of specific and effective treatments, have prompted great efforts by the medical community. Clinical, radiological, and autopsy reports of pulmonary fibrosis were common after SARS and MERS, and the current evidence suggests that, in SARS-CoV-2 infection cases, pulmonary fibrosis can also complicate the clinical outcomes [8].

In these circumstances, due to the high number of COVID-19 cases, healthcare systems are facing increasing challenges with more patients experiencing post-COVID-19 pulmonary fibrosis and its consequences, including an increased risk of mortality, restrictive ventilatory dysfunction, and a reduced exercise capacity. At present, in the wake of the COVID-19 pandemic, we find ourselves grappling with a new facet of this condition—pulmonary fibrosis that emerges as a consequence of COVID-19 infection. This raises a critical question: how do we address the unique challenges posed by post-COVID-19 pulmonary fibrosis in the post-pandemic era?

This article aims to shed light on the specialized therapeutic approaches that have emerged to tackle pulmonary fibrosis in the post-COVID-19 era. We not only explore the established treatments for pulmonary fibrosis, but also the innovative and personalized strategies being developed to improve patient outcomes. As we delve into the evolving landscape of pulmonary fibrosis treatment, we find reasons for hope and optimism, underpinned by the tireless efforts of researchers and healthcare providers. In this article, we take a closer look at these therapeutic approaches and the potential avenues for future developments in the management of post-COVID-19 pulmonary fibrosis. Additionally, we investigate the risk of developing post-COVID-19 pulmonary fibrosis in terms of the pathogenic mechanisms involved and the potential effect of available therapies to minimize this risk.

## 2. Materials and Methods

We conducted a systematic literature review to identify the relevant articles published between January 2000 and November 2023. The search was performed in PubMed using keywords, such as [pulmonary fibrosis], AND ([ARDS] OR [MERS] OR [COVID-19] OR [SARS]). Additionally, we manually screened the reference lists of identified articles for additional relevant studies. The initial screening involved assessing titles and abstracts to exclude irrelevant studies. The inclusion criteria comprised articles that explored therapeutic approaches for pulmonary fibrosis in the context of post-COVID-19 complications. After reviewing 2312 full-text articles for eligibility, 413 articles were ultimately selected for thorough analyses. Two authors independently extracted the data from the selected articles. The extracted information included study design, patient demographics, types of therapeutic interventions, and reported outcomes. Any discrepancies in the data extraction were resolved through a consensus between these authors. Our analysis was structured around key therapeutic categories, including pharmacological interventions, rehabilitation strategies, and emerging therapies. Each category was further examined in terms of the reported efficacy and safety.

## 3. Pathophysiology of Post-COVID-19 Pulmonary Fibrosis

Fibrosis and organ failure contribute to approximately one-third of global deaths [9]. Consequently, fibrosis emerges as a compelling research focus for developing novel antifibrotic therapeutic agents or for impeding the progression of excessive inflammatory processes, as observed in certain COVID-19 patients.

Fibrosis represents the ultimate outcome of nearly all chronic inflammatory diseases and can be viewed as a consequence of a disrupted wound healing process, directly linked to the severity of an acute event [10]. Various pathways contributing to lung injury in COVID-19 patients, involving both viral and immune-mediated mechanisms, have been elucidated [11]. The studies indicate that COVID-19-induced bilateral interstitial pneumonia affects the pulmonary alveolar epithelium, leading to the deposition of excess collagen (fibrosis) [12,13].

The mechanisms of pulmonary fibrosis have been extensively studied, and antifibrotic drugs have already been recommended, particularly for idiopathic pulmonary fibrosis (IPF) and progressive fibrosis [14,15]. The factors associated with the poor prognosis of COVID-19 patients, such as older age, comorbidities, and smoking, align with similar risk factors observed in IPF patients, predominantly affecting male smokers over the age of 60 years with cardiovascular disease [16,17]. Many IPF patients are currently treated with antifibrotic drugs, like nintedanib and pirfenidone, as well as those in the investigational phase (buloxibutid and saracatinib), demonstrating a decrease in the rate of lung function decline [18,19].

Given the relatively short duration since the onset of the COVID-19 pandemic and the predominant focus on acute case management, there is insufficient data to assess the potential risks and benefits of initiating antifibrotic therapy in patients with post-infection pulmonary sequelae [20]. Antifibrotic drugs may prove beneficial in treating or preventing fibrosis, offering relief to patients experiencing persistent issues or those who have undergone treatment but still grapple with fibrotic lung disease, potentially avoiding severe or fatal COVID-19-related complications [21].

**Figure 1:**
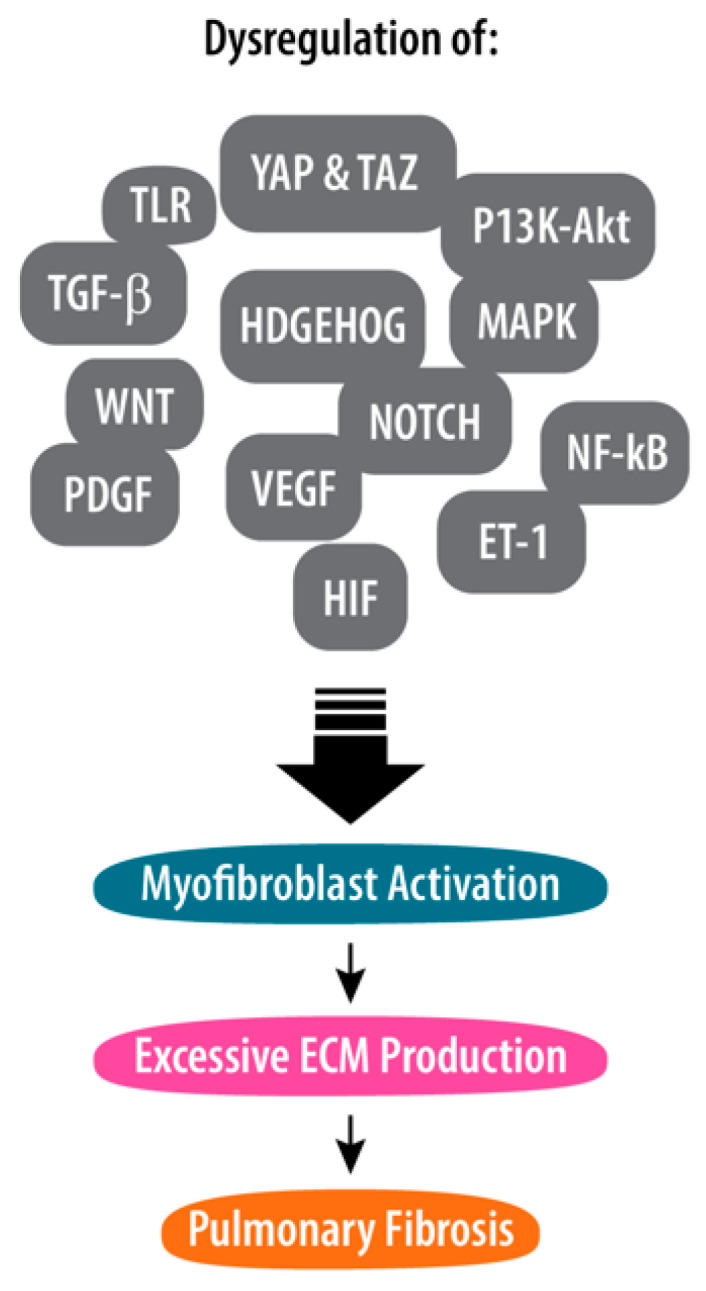
Signal pathways linked to post-COVID-19 pulmonary fibrosis pathophysiology. Legend: YAP—yes-associated protein 1; TAZ—transcriptional coactivator with PDZ-binding motif; TLR—toll-like receptor; TGF-β—transforming growth factor-beta; MAPK—mitogen-activated protein kinase; WNT—wingless/Int; PDGF—platelet-derived growth factor; VEGF—vascular endothelial growth factor; NF-κB—nuclear factor-kappa B; ET-1—endothelin-1; HIF—hypoxia-inducible factor.

Various signal transduction pathways are implicated in the pathophysiology of post-COVID-19 pulmonary fibrosis (Figure 1).

1. Toll-like receptor (TLR) signaling is integral to the innate immune response, with TLRs on immune cells recognizing viral components during SARS-CoV-2 infection. TLR activation triggers a cascade leading to immune responses, including pro-inflammatory cytokine and interferon production [22].
2. Transforming growth factor-beta (TGF-β) plays a pivotal role in post-COVID-19 lung fibrosis by stimulating fibroblast activation, promoting the transition to myofibroblasts, and inducing excessive extracellular matrix production. TGF-β signaling can also trigger the epithelial–mesenchymal transition, causing epithelial cells to acquire mesenchymal characteristics [23].
3. The wingless/Int (WNT) signaling pathway, a complex system vital for cellular processes, can be activated during tissue repair after COVID-19-induced lung damage. WNT ligands, released during repair, bind to receptors, initiating signaling events, including the stabilization and nuclear translocation of β-catenin [24].
4. Yes-associated protein 1 and transcriptional coactivator with PDZ-binding motif, transcriptional coactivators crucial for gene expression regulation, can, when active, prompt fibroblast activation, myofibroblast differentiation, and excessive extracellular matrix production [25].
5. The Hedgehog signaling pathway, initiated by Hedgehog ligands, like Sonic Hedgehog, Indian Hedgehog, and Desert Hedgehog, can be triggered during the repair of tissues associated with COVID-19. The binding of these ligands to cell surface receptors triggers intracellular signaling, leading to the translocation of Gli transcription factors into the nucleus for gene expression regulation [26].
6. The NOTCH signaling pathway is crucial for cell communication and various developmental processes. Following a COVID-19 infection, there is a potential for the expression of NOTCH receptors and ligands during the process of tissue repair. Activation occurs through receptor–ligand interactions, leading to fibroblast activation and differentiation into myofibroblasts [27,28].
7. The PI3K-Akt signaling pathway, activated by cell surface receptors, like receptor tyrosine kinases or G protein-coupled receptors, promotes cell survival by converting phosphatidylinositol 4,5-bisphosphate to phosphatidylinositol 3,4,5-trisphosphate (PIP3). PIP3 acts as a secondary messenger activating Akt, a serine/threonine kinase. The dysregulation of this pathway in post-COVID-19 lung fibrosis can lead to fibroblast activation, excessive extracellular matrix production, and enhanced cell survival [29,30].
8. The mitogen-activated protein kinase (MAPK) signaling pathway activates MAPKs, such as ERK, JNK, and p38. MAPK signaling, triggered by oxidative stress, can release pro-fibrotic factors, contributing to fibrogenesis. Notably, MAPK signaling is linked to the epithelial–mesenchymal transition process, further associating it with tissue remodeling in pulmonary fibrosis post-COVID-19 [31,32].
9. The nuclear factor-kappa B (NF-κB). Post-COVID-19-activated NF-κB signaling promotes an inflammatory microenvironment in the lungs by inducing pro-inflammatory cytokines, chemokines, and adhesion molecules. Additionally, NF-κB signaling activates fibroblasts and matrix metalloproteinases (MMPs), contributing to extracellular matrix degradation [33,34].
10. Platelet-derived growth factor (PDGF), comprising various isoforms and receptors, can be discharged as part of tissue repair processes. PDGF signaling activates fibroblasts, recruits fibroblast progenitor cells, and stimulates the excessive production of extracellular matrix components, including collagen. Additionally, PDGF contributes to angiogenesis and vascular remodeling, processes associated with fibrosis [35].
11. Vascular endothelial growth factor (VEGF). In post-COVID-19 lung fibrosis, VEGF plays a dual role. While it supports tissue repair through angiogenesis, excessive VEGF activity can lead to disorganized and leaky blood vessels, contributing to fibrosis. VEGF-A also stimulates fibroblast activation, proliferation, and immune cell recruitment, further contributing to tissue remodeling and the inflammatory response in the lungs [35].
12. Endothelin-1, a peptide regulating blood vessel constriction and blood pressure, can contribute to fibrosis. Excessive vasoconstriction in affected areas can increase tissue damage, influence the inflammatory response by recruiting immune cells and releasing pro-inflammatory mediators, and impact tissue remodeling through the effects on fibroblasts and extracellular matrix production [36,37].
13. Hypoxia-inducible factor (HIF) is a crucial transcription factor responding to low oxygen levels. Composed of HIF-1α (oxygen-sensitive) and HIF-1β (constitutively expressed) subunits, HIF-1α stabilizes and translocates to the nucleus under hypoxia, activating genes for adapting to low oxygen. In post-COVID-19 lung fibrosis, the HIF pathway contributes to fibroblast activation, angiogenesis, and inflammation, influencing myofibroblast differentiation, extracellular matrix production, blood vessel formation, and inflammatory response modulation [38,39].
14. Connective tissue growth factor (CTGF) is a multifunctional protein that regulates cell adhesion, migration, proliferation, and survival; promotes the production of extracellular matrix components, such as collagen, fibronectin, and proteoglycans; mediates interactions between cells and the extracellular matrix; and plays an essential role in tissue repair, fibrosis, and the modulation of growth factor responses [40,41].

## 4. Post-COVID-19 Pulmonary Fibrotic Process Progression

The other two strains of the coronavirus family that have been considered highly pathogenic in previous years, SARS-CoV and MERS-CoV, are genetically related to SARS-CoV-2 and cause similar lung diseases [42]. Similar to SARS-CoV, SARS-CoV-2 binds to human cells using angiotensin-converting enzyme 2 (ACE2) as a receptor. MERS-CoV uses dipeptidyl peptidase 4 for viral entry [43]. A multisystem damage explanation is supported by the presence of the ACE2 receptor in many tissues, such as the lungs, kidneys, heart, and intestine [44,45].

Longer follow-up data over a 15-year period showed that there were interstitial abnormalities in 4.6% of patients infected with SARS-CoV [46]. Although patients who recovered from MERS were less well-described in the literature, there was evidence of fibrotic lesions in about one third of patients with chest radiographs after discharge [47]. We can thus predict that, in the case of SARS-CoV-2, the progression can be comparable. Until the data are available remotely, it is important to consider the experience of previous coronavirus outbreaks. In addition, post-inflammatory pulmonary fibrosis has been recognized and entered into disease coding systems, confirming the frequency of cases in patients with MERS [48].

In the acute phase, the lower lobes are the primary site for bilateral ground glass opacities with or without consolidations in SARS-CoV-2 infected patients [49]. However, it should be considered that long-term lung injury, especially fibrotic interstitial lung disease, can occur after the virus is eliminated. The degree of reticulation evidenced on computed tomography (CT) scans correlates with the quality of life measures and lung functions that indicate a pattern of restriction, such as forced vital capacity (FVC) and diffusing capacity of the lungs for carbon monoxide (DLCO) [46].

As a fibro-proliferative disease with a genetic addiction, pulmonary fibrosis is linked to prolonged inflammation. The factors contributing to the pathophysiology of post-COVID-19 pulmonary fibrosis are described above. Due to their intense inflammatory processes, there is an ongoing release of these mediators, which can lead to inappropriate remodeling and fibrosis [50,51]. Although the current data suggest the influence of the thrombotic process as a precursor of pulmonary and hepatic fibrosis [52,53], no possibilities of therapeutic interventions currently exist due to the difficulty of separation from physiological recovery processes [54,55].

Thille and colleagues described in a cohort of 159 autopsies of patients with ARDS that 4% of patients with a disease duration of less than 1 week, 24% of patients with a disease duration between 1 and 3 weeks, and 61% of patients with a disease duration longer than 3 weeks, developed pulmonary fibrosis [56]. This report and other data indicate that pulmonary fibrosis occurs in the early stages of acute respiratory distress syndrome (ARDS) [57]. In a 12-month follow-up study of SARS patients, 67 (21.5%) had pulmonary fibrotic alterations, while 85 of 311 patients (27.3%) had lung diffusion abnormalities (DLCO 80% predicted) within 65 days after discharge [58].

Fibrotic changes were seen in 33.9% of patients in a study that included 62 patients, and this finding was more prevalent in late-disease (8–14 days after symptom onset) rather than in early disease (7 days after symptom onset) situations [59]. The detection of fibrotic changes during the early stages of the disease suggests an attempt to repair lung damage. However, it is still too early in the disease process to assess whether this finding can be resolved with time or with progression to fixed pulmonary fibrosis [60].

## 5. Current Therapeutic Approaches

In the aftermath of the COVID-19 pandemic, exploring effective therapeutic approaches for post-COVID-19 fibrosis has become imperative, aiming to mitigate the long-term pulmonary consequences and enhance the quality of life for affected individuals. Several therapeutic approaches are being explored for post-COVID-19 fibrosis. These are listed in Table 1 and presented below.

Antifibrotic therapy, in the context of post-COVID-19 fibrosis, represents a nuanced approach aimed at interrupting pathogenic fibrotic processes triggered by SARS-CoV-2. From deciphering the intricate molecular pathways that drive fibrosis to evaluating the efficacy of novel drugs, this specialized exploration is navigating the forefront of scientific endeavors. Currently recommended antifibrotic therapies for idiopathic pulmonary fibrosis and progressive pulmonary fibrosis based on nintedanib and pirfenidone (only for idiopathic pulmonary fibrosis) have been observed to slow down the progression of the disease [61,62]. Since both molecules do not act by suppressing immunity, there is no cause for concern about their use for viral infections.

### 5.1. Pirfenidone Is Categorized as a Pyridone, with the Following Points Outlined as Its Mechanisms of Action (Figure 2)

#### 5.1.1. Inhibition of TGF-β

Pirfenidone inhibits the TGF-β signaling pathway, a crucial mechanism in pulmonary fibrosis. By reducing TGF-β production and interfering with downstream effectors, like Smad proteins, pirfenidone mitigates fibrotic processes, specifically reducing collagen production and preventing excessive scar tissue deposition [63].

**Figure 2:**
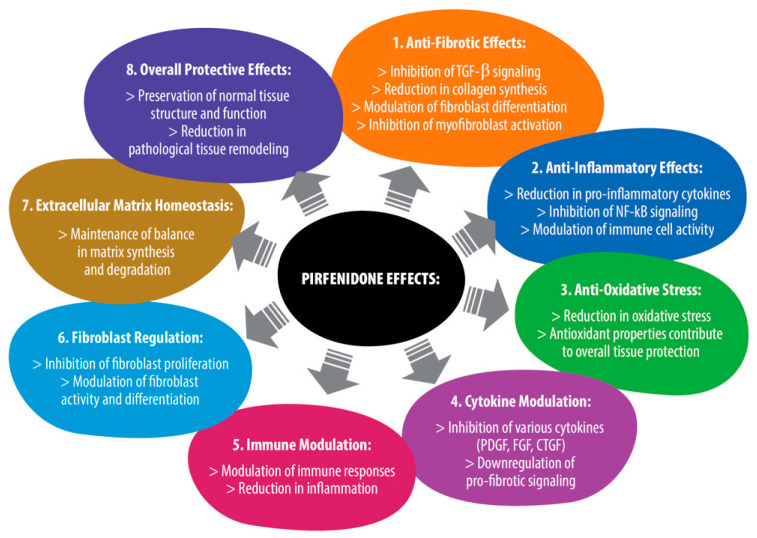
The mechanisms of action of pirfenidone.

#### 5.1.2. Anti-Inflammatory Effects

Pirfenidone reduces pro-inflammatory cytokines, like tumor necrosis factor-α and interleukins, by potentially interfering with NF-κB activation and TGF-β. It can modulate signaling cascades, including those involving NF-κB and MAPK, and mitigate oxidative stress, indirectly affecting interleukin (IL)-6 production. This multifaceted action contributes to the reduction in IL-6 levels, making pirfenidone a potential treatment option for COVID-19 by dampening the inflammatory response and addressing fibrotic triggers [64].

#### 5.1.3. Fibroblast Modulation

Pirfenidone’s direct inhibition of fibroblast proliferation and activation is crucial in its anti-fibrotic mechanism for post-COVID-19 pulmonary fibrosis. It modulates fibroblast differentiation, especially into myofibroblasts, known for excessive extracellular matrix protein production. The molecule regulates the fibrotic response by interfering with signaling pathways of key cytokines involved in fibroblast activation, including PDGF, fibroblast growth factor (FGF), and CTGF [63,65].

#### 5.1.4. Reduction in Oxidative Stress

Acting as an antioxidant, pirfenidone neutralizes reactive oxygen species, inhibits lipid peroxidation, and upregulates antioxidant enzymes, like superoxide dismutase and catalase. This contributes to maintaining the cellular redox balance, protecting against oxidative damage, and potentially preserving the mitochondrial function [66].

#### 5.1.5. Modulation of Matrix Metalloproteinases (MMPs)

This agent modulates MMPs, crucial for extracellular matrix breakdown. It potentially influences the MMP tissue inhibitor of metalloproteinase balance, promoting reduced MMP activity and maintaining a controlled synthesis-degradation equilibrium in the extracellular matrix. This regulation helps prevent excessive matrix deposition and supports controlled tissue turnover outcomes [67].

#### 5.1.6. Downregulation of Pro-Fibrotic Mediators

This drug reduces CTGF expression, a key fibrotic player. Inhibiting TGF-β signaling, it downregulates CTGF, a TGF-β downstream target. Modulating Smad proteins and mitigating inflammation, it limits fibroblast activation, decreasing cellular sources of CTGF. Its anti-fibrotic effects, including reduced collagen synthesis, attenuate CTGF-mediated extracellular matrix deposition [63].

In addition, it can have immunomodulatory effects, influencing the activity of immune cells involved in inflammation and tissue repair. By modulating the immune response, pirfenidone contributes to the overall reduction in fibrotic processes [68].

**Table 1:** Comprehensive overview of therapeutic approaches for post-COVID-19 fibrosis.

Therapeutic Agent	Mechanisms of Action	Specific Actions in Post-COVID-19 Fibrosis	Clinical Findings
Pirfenidone	1. Inhibition of TGF-β 2. Anti-inflammatory effects 3. Fibroblast modulation 4. Reduction of oxidative stress 5. Modulation of MMPs 6. Downregulation of pro-fibrotic mediators 7. Immunomodulatory effects	- Reduces TGF-β production - Mitigates fibrotic processes - Directly inhibits fibroblast proliferation - Acts as an antioxidant - Modulates MMPs - Reduces CTGF expression - Potential immunomodu-latory effects	INBUILD study [69] suggests nintedanib inhibits fibrogenesis in lung diseases, including SARS-CoV-2 infection. Inhaled Pirfenidone under assessment
Nintedanib	1. Tyrosine kinase inhibition 2. Inhibition of multiple receptors 3. Reduction in fibroblast activity 4. Anti-angiogenic effects 5. Anti-inflammatory effects	- Interferes with tyrosine kinases - Targets PDGF, FGF, and VEGF receptors - Reduces fibroblast activation - Exhibits anti-angiogenic effects - Attenuates IL-1β concentration	Demonstrated inhibition of fibrosis pathways in INBUILD study [69], including those relevant to SARS-CoV-2 infection
Corticosteroids	Suppression of inflammatory response	- Suppresses fibroblast activation - Reduces collagen synthesis - Helps regulate immune response - Improves lung function in post-COVID-19 ILD	Improvements in lung function and symptoms observed in ILD patients post-COVID-19 with corticosteroid treatment
Anti-interleukins	- IL-6 inhibition (e.g., tocilizumab) - IL-1 inhibition (e.g., anakinra)	- Potential modulation of inflammatory and fibrotic responses in post-COVID-19 complications	Ongoing research to explore the impacts of IL-6 and IL-1 inhibition on post-COVID-19 fibrosis
Immunosuppressants	Tacrolimus, cyclosporine, and mycophenolate mofetil inhibit the proliferation of immune cells	- Modulation of immune response - Potential steroid-sparing option for treatment	Mycophenolate mofetil showed improvements in FVC and DLCO in post-COVID-19 ILD, emphasizing the need for further research
Additional therapies	- Buloxibutide (AT2R agonist) - Saracatinib (Src kinase inhibitor) - Sirolimus (immunosuppressant) - Resveratrol (anti-inflammatory)	- Stabilizing effect on FVC (buloxibutide) - Modulation of fibroblast activity (saracatinib) - Reduction in pulmonary fibrosis (sirolimus, resveratrol)	Ongoing studies indicate potential benefits of these therapies for post-COVID-19 fibrosis

Legend: TGF-β—transforming growth factor-beta; MMPs—matrix metalloproteinases; CTGF—connective tissue growth factor; SARS-CoV-2—severe acute respiratory syndrome-2; COVID-19—coronavirus-19 disease; PDGF—platelet-derived growth factor; FGF—fibroblast growth factor; VEGF—vascular endothelial growth factor; IL—interleukin; ILD—interstitial lung disease; FVC—forced vital capacity; DLCO—diffusing capacity of the lungs for carbon monoxide; AT2R—angiotensin II type-2 receptor.

Researchers are assessing an inhaled version of pirfenidone in COVID-19 patients. The INBUILD study concluded that nintedanib therapy appeared to inhibit fibrogenesis in a wide range of lung diseases, including, due to the similar pathogenic pathways, SARS-CoV-2 infection [69].

### 5.2. The Mechanisms of Action of Nintedanib

Nintedanib is a small molecule that acts as a tyrosine kinase inhibitor, specifically targeting multiple receptors involved in the fibrotic process. The mechanisms of action of nintedanib are the following:

#### 5.2.1. Tyrosine Kinase Inhibition

Nintedanib interferes with the activity of specific enzymes called tyrosine kinases. Tyrosine kinases play a role in cellular signaling, and their dysregulation is associated with the development of fibrosis [70].

#### 5.2.2. Inhibition of Multiple Receptors

Nintedanib targets and inhibits multiple receptors involved in fibrosis, including receptors for PDGF, FGF, and VEGF. These growth factors are implicated in the proliferation and activation of fibroblasts, which contribute to the excessive production of collagen and other extracellular matrix components observed in fibrosis [71].

#### 5.2.3. Reduction in Fibroblast Activity

By inhibiting the signaling pathways triggered by PDGF, FGF, and VEGF, Nintedanib reduces the activation and proliferation of fibroblasts. Fibroblasts are key cellular players in the fibrotic process, contributing to the deposition of scar tissue [71].

#### 5.2.4. Anti-Angiogenic Effects

Nintedanib’s inhibition of VEGF receptors also has anti-angiogenic effects, meaning it interferes with the formation of new blood vessels. This can impact the vascular component of fibrotic tissue and help manage fibrosis [72,73].

#### 5.2.5. Anti-Inflammatory Effects

Nintedanib has been shown to attenuate IL-1β concentration in bronchoalveolar lavage [74,75].

Both pirfenidone and nintedanib can be associated with hepatotoxicity, and liver dysfunction is common in patients infected with SARS-CoV-2. Among the individuals with confirmed COVID-19, 22% (168 out of 757) exhibited elevated liver enzyme values, while 39% (56 out of 142) of those with severe illnesses also had elevated levels [76].

Given the variable mix of immunologically mediated lesions and classical acute lung injuries observed in patients with SARS-CoV-2 infections, it is reasonable to presume that antifibrotic therapy used should be individualized to specifically address different pathogenic pathways. Alternatively, it can include combinations of molecules that can exert broader inhibitory effects on both patterns.

### 5.3. Corticosteroids

Corticosteroids help suppress the inflammatory response, reducing the release of pro-inflammatory cytokines and mitigating inflammation. In cases of post-COVID-19 fibrosis, an overactive immune response can contribute to tissue damage, and corticosteroids help regulate this response [19,77]. These can inhibit fibroblast activation and reduce collagen synthesis, helping to limit the progression of fibrosis. Corticosteroids can help suppress the scarring process by interfering with the deposition of collagen and other fibrotic components in the affected tissues. This can contribute to preventing or reducing the severity of fibrotic changes. In addition, these can help prevent or mitigate airway remodeling by suppressing inflammation and the associated tissue changes. According to a study, lung function improved when a subset of individuals with severe interstitial lung disease (ILD) received corticosteroids [78]. Treatment was initiated on the 61st day (with a range of ±19 days) after discharge, and the patients received a maximum initial dose of 0.5 mg/kg of prednisolone. Considering the probable absence of a sustained inflammatory trigger, an average starting dose of 26.6 mg was administered with a rapid 3-week taper. The several key characteristics of patients with persistent ILD post-COVID-19 were identified. They were mostly male (71.5%) and overweight, with an average body mass index of 28.3, and 26% were obese. Of these, the majority had at least one co-morbidity, the most common being diabetes and asthma (22.9%). The average length of hospital stay was 16.9 days, 82.9% required oxygen, 55% were in intensive care, and 46% required invasive mechanical ventilation. Treatment resulted in a mean increase in transfer factor of 31.6% (*p* < 0.001) and forced vital capacity of 9.6% (*p* = 0.014), along with a significant improvement of symptoms and lung imaging appearance.

## 6. Emerging Therapies and Research

### 6.1. Anti-Interleukin Therapies

In the context of post-COVID-19 fibrosis, researchers have been interested in understanding the role of interleukins and exploring the potential benefits of anti-interleukin therapies. IL-6 is known to be involved in the inflammatory response, and elevated levels of IL-6 have been observed in some COVID-19 patients [79]. Therapies targeting IL-6, such as tocilizumab, have been investigated in the management of severe COVID-19 cases with systemic inflammation. IL-6 is generally considered to be a profibrotic molecule; an experimental study with the bleomycin model of pulmonary fibrosis suggested that the inhibition of IL-6 in the early phase of lung injury promoted fibrosis and that inhibition in the later stages of injury at the onset of the fibrotic phase could ameliorate fibrosis [80,81,82,83]. Also, several studies have shown that protective lung ventilation tends to decrease radiographic abnormalities following ARDS [84].

IL-1 is another pro-inflammatory cytokine that plays a role in the immune response. Drugs, like anakinra, which inhibits IL-1, have been explored in various inflammatory conditions [85]. The research can investigate whether anti-interleukin therapies can be beneficial in addressing inflammation and fibrosis associated with post-COVID-19 complications.

### 6.2. Immunosuppressants

Severe cases of COVID-19 are associated with cytokine storms, where the immune system releases an excessive amount of pro-inflammatory cytokines [86]. This hyperinflammatory state can contribute to tissue damage, including fibrosis. Immunosuppressants can be considered to modulate this exaggerated immune response. Medications, like tacrolimus and cyclosporine, are calcineurin inhibitors that suppress the activity of T cells, a type of immune cell [87,88]. These drugs are used to treat various autoimmune conditions and can be considered in certain post-COVID-19 cases where there is an abnormal immune response. Mycophenolate mofetil is an immunosuppressant that inhibits the proliferation of immune cells, including T and B cells. It is used in conditions where there is an overactive immune response. One study [89] described patients with organizing pneumonia (OP) post-COVID-19 in the pre-vaccination era with inadequate clinical responses to corticosteroids, but improved after the addition of mycophenolate mofetil (MMF). MMF, known for its immunosuppressive properties, was administered after patients experienced suboptimal responses to high-dose corticosteroids and exhibited steroid toxicity. For the two patients included in the study, to evaluate tolerance, MMF was administered at 500 mg twice daily (BID) and raised to 1 g BID after two weeks, and maintained for 6 months only for the former. Despite the mild side effects from MMF, both patients showed a gradual improvement in FVC, DLCO, and stabilization during the course of treatment, allowing for the reduction in and eventual discontinuation of corticosteroid therapy. The use of MMF in post-COVID-19 ILD patients with OP suggests a potential alternative or steroid-sparing option for treatment, emphasizing the need for further research and clinical trials in this area.

### 6.3. Additional Fibrotic Therapy

As previously mentioned, the TGF-β pathway was proposed as a model for the development of post-COVID-19 fibrosis. In this regard, there are a number of molecules in the research that can be used in the therapy of these cases, including TD139—an inhaled small-molecule inhibitor of galectin-3 [90].

Mesenchymal stem cells (MSCs), having a multipotent capacity to replace damaged alveolar epithelium, secrete anti-inflammatory factors, inhibit fibro-proliferation, and are therefore considered for the treatment of ARDS, the main cause of mortality in COVID-19 diseases [91]. The use of mesenchymal stem cells is currently being evaluated in clinical trials [92]. The main arguments for the use of MSCs for SARS-CoV-2 infections are their ability to prevent excessive cytokine release and promote endogenous repair [93].

### 6.4. Future Opportinities

In the dynamic field of medical research, the pursuit of innovative therapeutic strategies has uncovered four promising molecules: buloxibutid, saracatinib, sirolimus, and resveratrol.

Regarding the angiotensin II type-2 receptor (AT2R) pathway and the stimulation of the renin–angiotensin system, two significant pathways implicated in the entry of the SARS-CoV-2 virus and the initiation of the host’s response [94], recent research has evaluated the impact of buloxibutide, an AT2R agonist, on pulmonary fibrosis patients [95]. While the study is ongoing, with the first results estimated to appear in March 2024, an interim analysis reveals a stabilizing effect and even an improvement of the FVC. Buloxybutide demonstrates a favorable safety profile, with no notable gastrointestinal issues or serious side effects reported. This low-molecular-weight, orally administered molecule has already been studied in hospitalized SARS-CoV-2 patients, showing a lower proportion needing extra oxygen after seven days of therapy compared to the placebo group [96].

Another promising molecule is saracatinib, offering hope by modulating fibroblast activity and inhibiting collagen deposition. Originally used in cancer therapies, saracatinib, a Src kinase inhibitor, alters TGF-β, subsequently suppressing human lung fibroblasts [97]. This action reverses fibrogenic pathways, including the epithelial–mesenchymal transition, immune responses, and extracellular matrix organization.

An ongoing study aims to explore the effectiveness of sirolimus therapy in reducing pulmonary fibrosis in COVID-19 hospitalized patients [98]. Sirolimus, an immunosuppressant used in post-transplant therapy with associated serious side effects, including pulmonary issues, was orally administered to inpatients. Treatment effects were evaluated through a 12-week follow-up session, focusing on a 10% threshold for pulmonary fibrosis evident in CT scans. Other endpoints included oxygen use and the impact on respiratory function. Sirolimus, known for its anti-proliferative effects on lymphoid and non-lymphoid cells, demonstrated beneficial effects in treating liver fibrosis [99] and lung conditions [100]. Recent studies indicate a significant decrease in the concentration of total fibrocytes, including CXCR4+ fibrocytes, after a 6-week sirolimus therapy [101]. Short-term sirolimus treatment has proven effective in reducing circulating fibrocytes with acceptable tolerability compared to a placebo.

Recent evidence for resveratrol, a molecule present in various plants with anti-inflammatory and antioxidant properties, reveals its ability to reduce the concentration of pro-inflammatory cytokines, thereby alleviating excessive inflammation and pulmonary fibrosis. These effects result from the inhibitions of TGF-β/Smad2/3/4, NF-κB, and Janus kinase/signal transducer and activator of transcription signal transduction pathways [102]. A clinical trial [103] is currently evaluating the therapeutic antifibrotic effects of resveratrol on patients discharged with COVID-19. Participants will undergo six months of treatment (1 g, orally, once daily) with a three-month follow-up, totaling a 9-month trial duration. The current data suggest that resveratrol can impede the progression of certain autoimmune diseases and presents a positive therapeutic prospect in pulmonary fibrosis [104]. Experimental studies have demonstrated its effectiveness in alleviating interstitial lung diseases by inhibiting Smad and Smad7 expressions, lung fibroblast proliferation and differentiation, and reducing collagen deposits [102,105].

## 7. Discussion

The COVID-19 pandemic has added a new dimension to the comprehension of pulmonary fibrosis. Although the acute respiratory impacts of SARS-CoV-2 are extensively documented, there is a mounting concern regarding the prolonged respiratory complications experienced by certain COVID-19 survivors. In the post-COVID-19 era, a growing number of individuals are receiving diagnoses of post-COVID-19 pulmonary fibrosis. This condition is thought to arise from the virus’s inflammatory response and the consequent damage to lung tissue, underscoring the necessity for specialized treatment approaches in the post-pandemic world.

Due to exaggerated inflammatory processes in the lung parenchyma, tissue remodeling exists, leading to the loss of alveolar and vascular structures [106]. Identifying molecules that can interfere with multiple pro-fibrotic and lung parenchymal remodeling mechanisms will facilitate the discovery of genuinely effective therapeutic principles for preventing or reversing post-COVID-19 pulmonary fibrosis.

While fibrotic processes were traditionally viewed as irreversible and progressive, the recent findings show that certain molecules, some already used for treating various types of pulmonary fibrosis, can slow down the decline and even stabilize lung function. There is currently hope that, based on preliminary studies, a selective AT2R agonist can reduce collagen deposition in the lungs, diminish vascular remodeling, and, when administered during the acute phase, decrease oxygen requirements in patients with SARS-CoV-2 infections. Although it is premature to speculate on the inclusion of this molecule in the evidence-based therapy, there are hopes that the link represented by the activation of the renin–angiotensin system can be therapeutically stabilized.

On the other hand, saracatinib, another molecule recently noted for its antifibrotic potential by influencing TGF-β, though not yet investigated in post-COVID-19 fibrosis, has demonstrated an increased potential in intercepting profibrotic inflammatory cascades and organizing the extracellular matrix [107].

The inflammatory pathway involving fibrocyte accumulation in the lungs via CXCR4 receptor coupling has been associated with the severity of COVID-19 [108]. The administration of sirolimus in these patients has proven to be a viable therapeutic option by reducing the number of circulating fibrocytes, with the validation of this hypothesis to be obtained from the results of a clinical trial currently underway [101].

Some promise for slowing down or even blocking the fibrotic lung process, both by modulating inflammation and by blocking collagen deposition in lung tissue, comes from a molecule found in large amounts in grapes or peanuts—resveratrol. Several studies confirm hopes for the use of this drug for interstitial lung diseases [109]. Resveratrol’s pathways of action are multiple, and the safety profile appears to be good. Since the doses used in the existing studies vary, further research is needed to determine the therapeutic dose and pharmacokinetics of this molecule.

These molecules, either in monotherapy or in combination, can represent potential pathways for reversing lung parenchymal damage in post-COVID-19 patients.

## 8. Conclusions

Antifibrotic therapies, such as pirfenidone and nintedanib, show promise in slowing down pulmonary fibrosis without compromising immunity. Pirfenidone targets TGF-β, has anti-inflammatory effects, and modulates fibroblasts, while nintedanib inhibits tyrosine kinases and reduces fibroblast activity. Corticosteroids also play a role in suppressing inflammation and fibrosis. The ongoing research explores novel therapies, including anti-interleukin treatments, immunosuppressants, and molecules, like buloxybutide, saracatinib, sirolimus, and resveratrol. These can offer diverse approaches to managing fibrosis. Emerging treatments, like mesenchymal stem cells and AT2R pathway modulation, are under investigation.

Furthermore, the initiation of any treatment demonstrating established anti-fibrotic efficacy in patients with COVID-19 should take place at the earliest feasible stage. This decision should be supported by clinical indicators or prognostic biomarkers that facilitate risk stratification for the progression to pulmonary fibrosis.

## Data Availability

All data included this article are available upon reasonable request.
